# Supplementary material for: PremPLI: a machine learning model for predicting the effects of missense mutations on protein-ligand interactions
Source: Commun Biol. 2021 Nov 19;4:1311. doi: 10.1038/s42003-021-02826-3 (PMC8604987; doi:10.1038/s42003-021-02826-3)
Supplement: Supplementary file 2 — Supplementary Information [file 42003_2021_2826_MOESM2_ESM.pdf]

PremPLI: a machine learning model for predicting the effects of missense mutations on protein-ligand Interactions

Tingting Sun[1#], Yuting Chen[1#], Yuhao Wen[1], Zefeng Zhu[1] and Minghui Li[1,*]

[1]Center for Systems Biology, Department of Bioinformatics, School of Biology and Basic Medical Sciences, Soochow University, Suzhou 215123, China

[#]These authors contributed equally

*corresponding author, minghui.li@suda.edu.cn

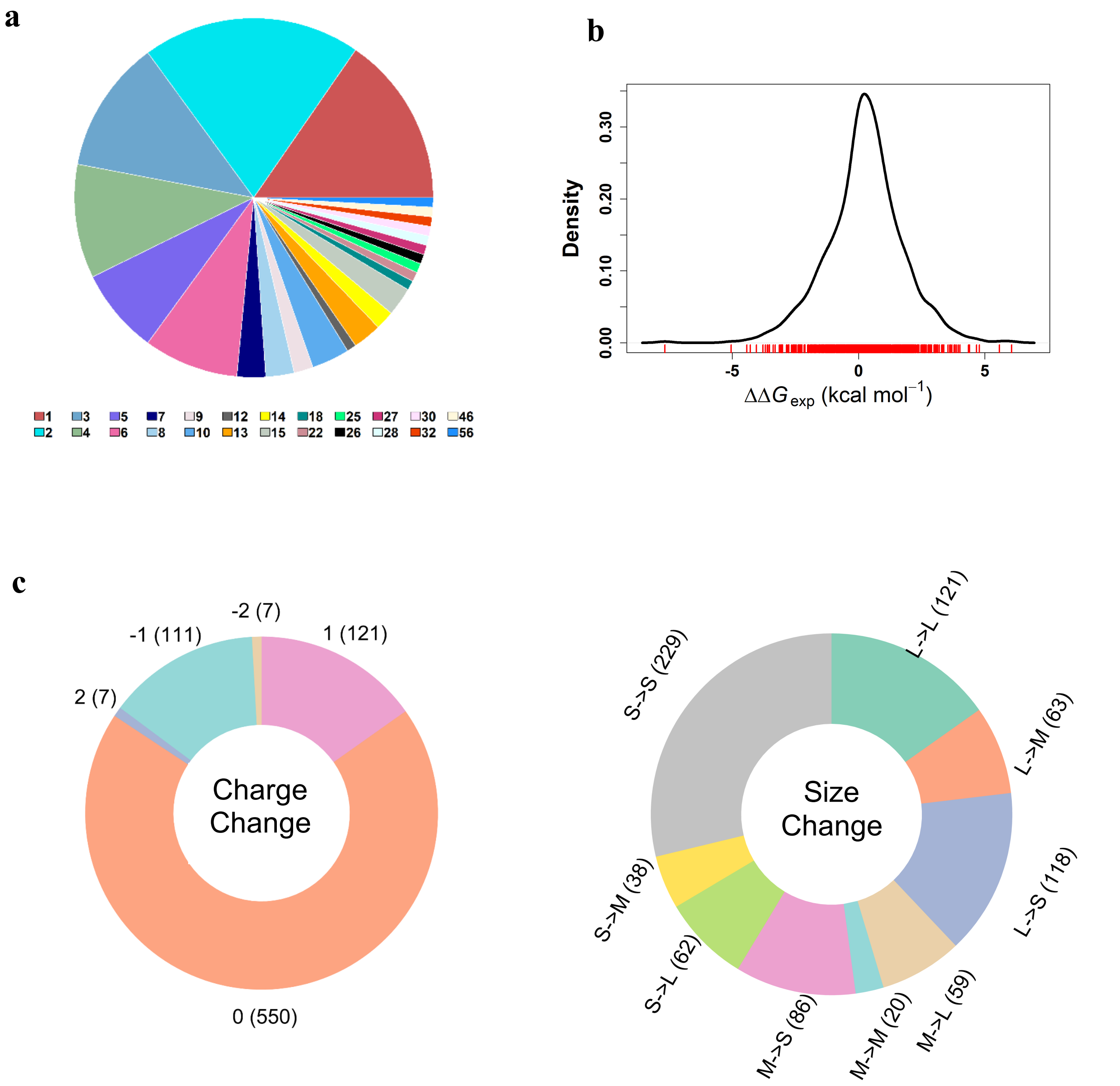

Supplementary Figure 1. S796 training set. (a) Distribution of the number of proteins across different number of mutations. (b) Distribution of experimental binding affinity changes $\Delta\Delta G_{exp}$. (c) Statistics of the types of mutations including charge and size changes of amino acids. Positive (K and R), negative (D and E), small (A, G, S, C, P, T, D and N), medium (V, H, E and Q), and large (I, L, M, K, R, F, W and Y) amino acids.

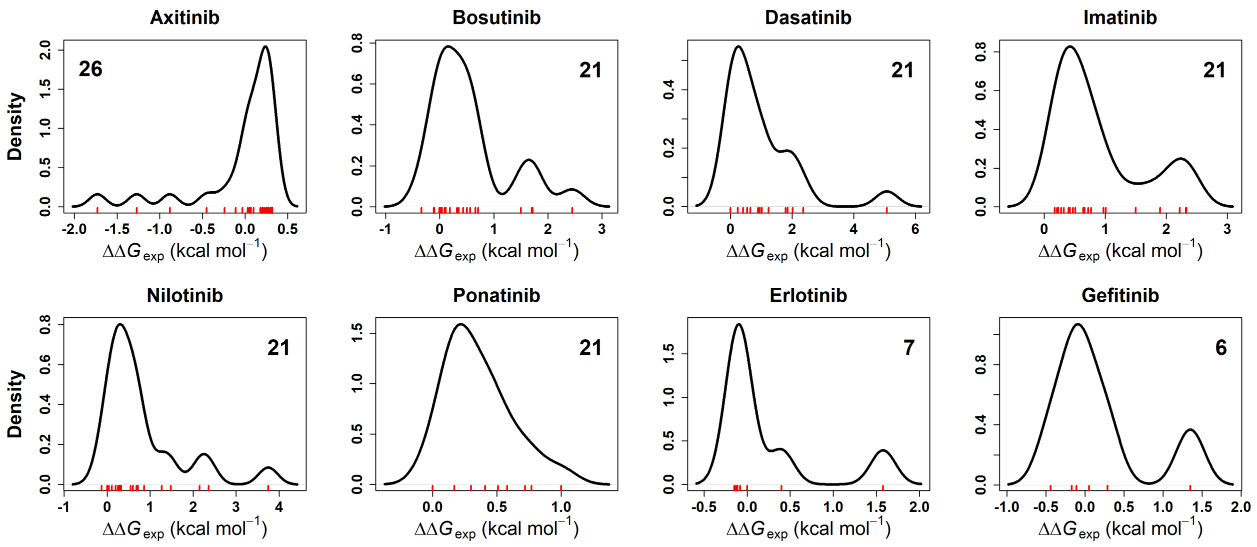

Supplementary Figure 2. Distribution of experimental binding affinity changes ($\Delta\Delta G_{exp}$) for each inhibitor in S144. The number of mutations for each type of inhibitor is shown on the density figure. Co-crystal structures of Abl bound to erlotinib or gefitinib were not available, so the docking models obtained from Hauser et al.[1] were used.

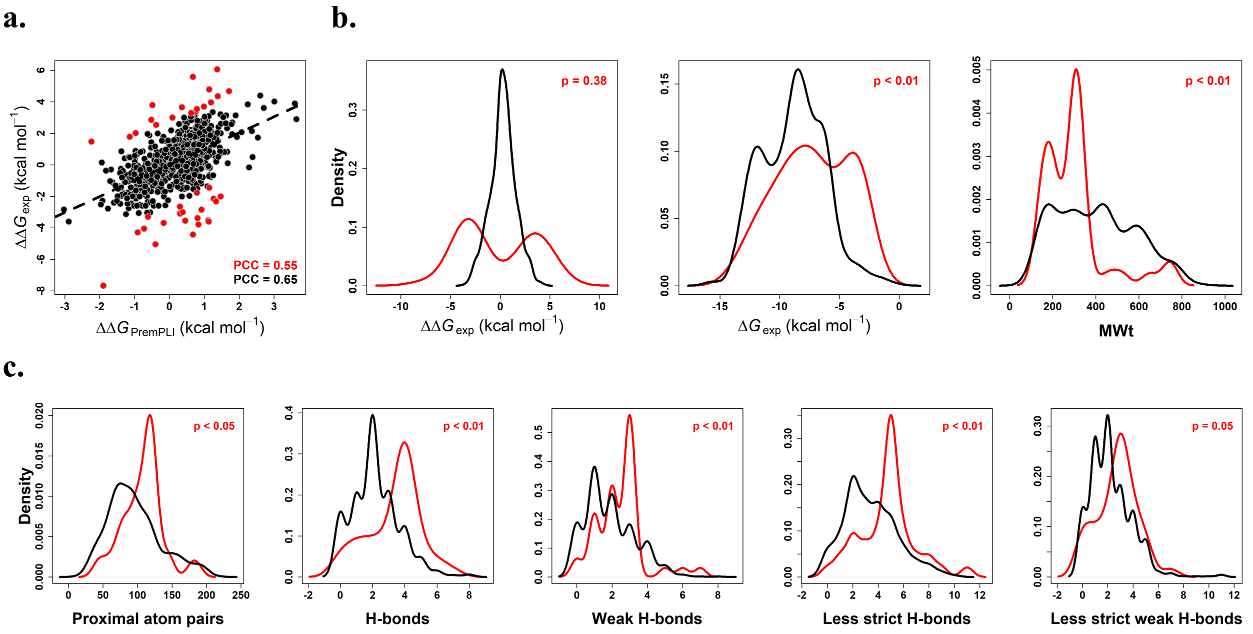

d.

Definition	5% outlier
$(\Delta\Delta G_{PremPLI} > 0$ & $\Delta\Delta G_{exp} > 1.36)/\Delta\Delta G_{exp} > 1.36$	0.67
$(\Delta\Delta G_{PremPLI} < 0$ & $\Delta\Delta G_{exp} <$ -1.36$)/\Delta\Delta G_{exp} <$ -1.36	0.27

Supplementary Figure 3. Outlier analysis of PremPLI. Leave-one-complex-out validation (CV3) results were used for these analyses. Black: all mutations except for 5% outliers; red: 5% outliers. (a) Pearson correlation coefficient between experimental and calculated changes in binding affinity. (b) Distibution of experimental binding affinity change ($\Delta\Delta G_{exp}$), experimental binding affinity (ΔG_{exp}) between wild-type protein and ligand, and molecular weight (MWt) of ligand. p-value (p) is shown indicating whether the difference between 5% outliers and the remaining mutations is statistically significant (t-test). (c) Distibution of the number of proximal atom pairs, hydrogen bonds (H-bonds), weak hydrogen bonds, less strict hydrogen bonds, and less strict weak hydrogen bonds formed between the mutated site and other atoms in the complex, repectively, which were calculated by Arpeggio[2]. (d) True positive rate.

a. S144

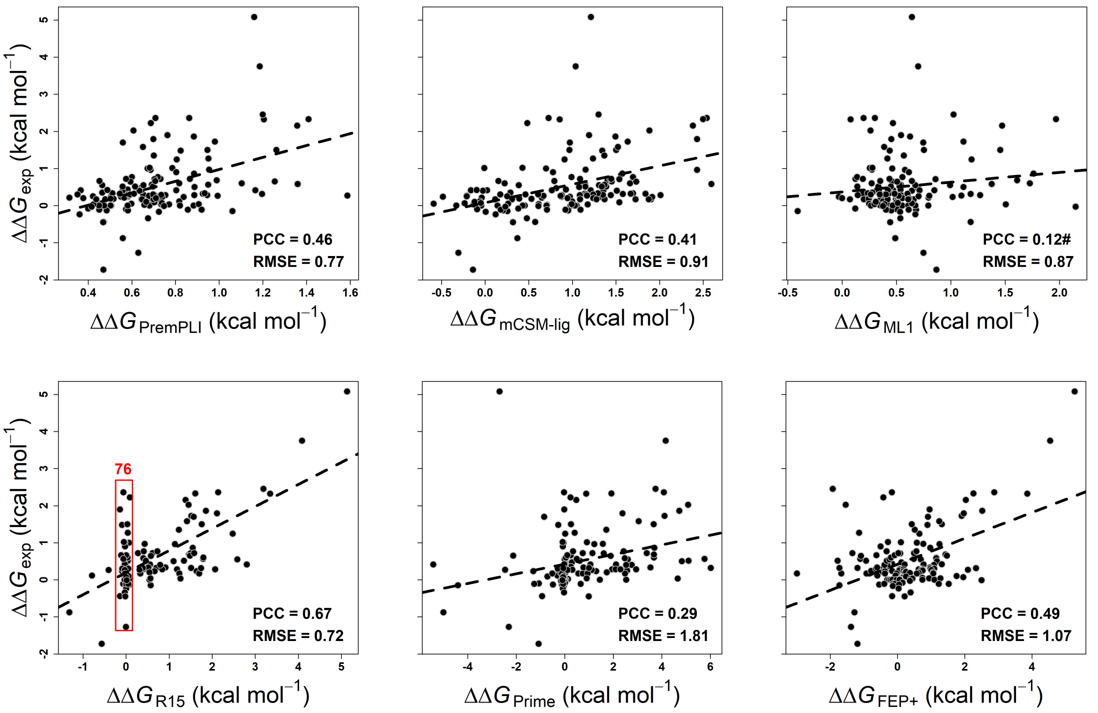

b. S99

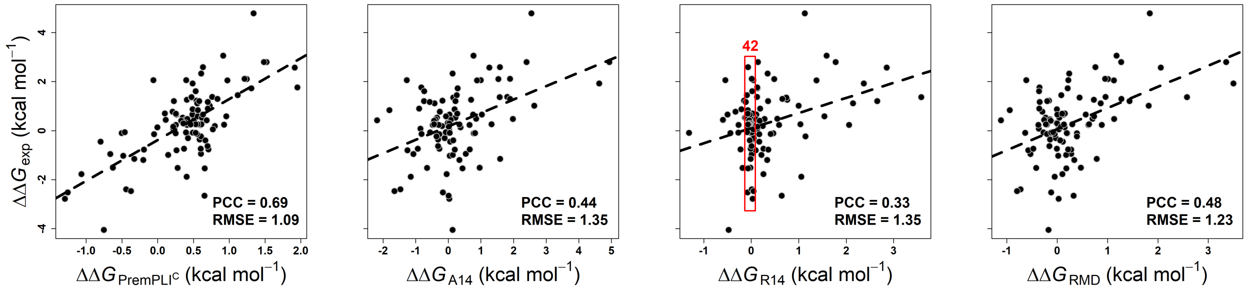

Supplementary Figure 4. Performance for different methods tested on S144 and S99 datasets. Pearson correlation coefficients (PCC) between experimental and calculated binding affinity changes for different methods tested on S144 (a) and S99 (b) datasets. All correlation coefficients are statistically significantly different from zero (p-value < 0.01, t-test) except $^{\#}p$-value = 0.14 for ML1. The prediction values of Rosetta for half of mutations in S144 (R15, 76 mutations in red box) and S99 (R14, 42 mutations in red box) are around zero.

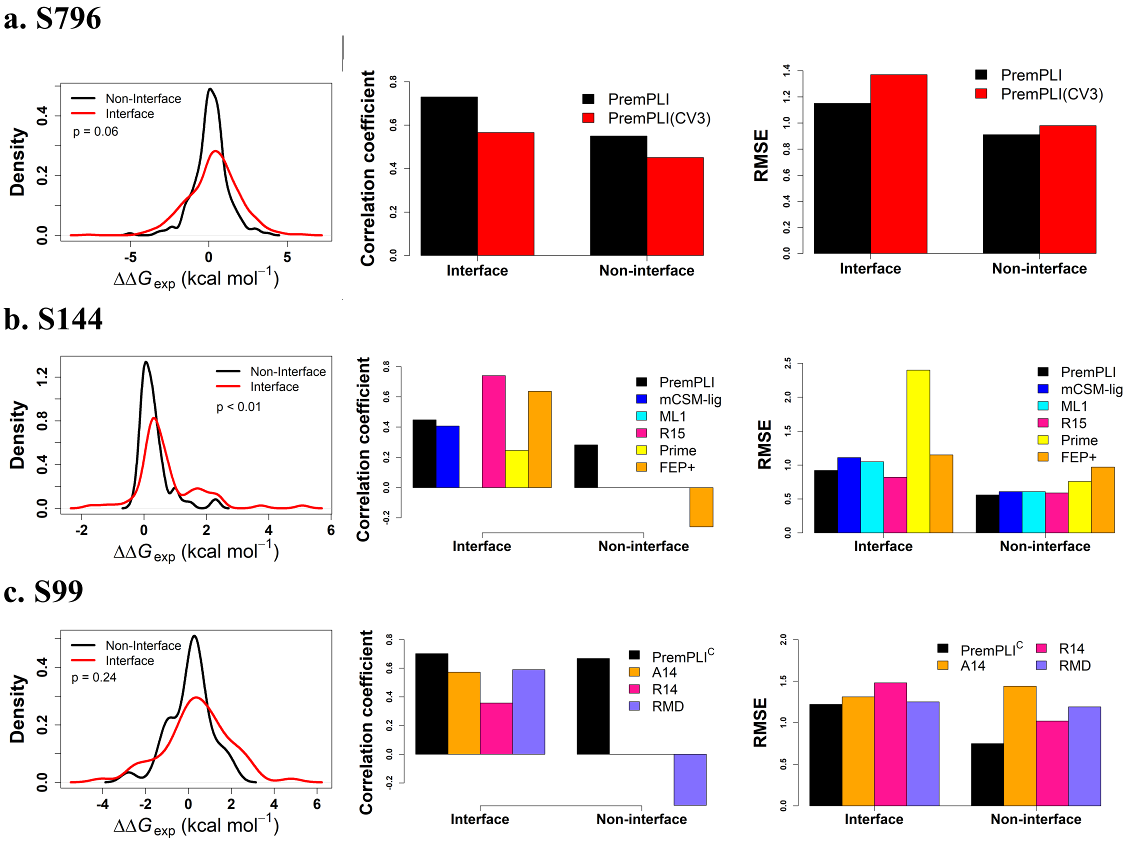

d.

Category	S796	S144	S99
**Interface**	545	75	67
**Non-interface**	251	69	32

Supplementary Figure 5. Analysis for interface and non-interface mutations. Distribution of experimental binding affinity changes, Pearson correlation coefficients, and root-mean-square errors for interface and non-interface mutations from S796 (a), S144 (b) and S99 (c) datasets, respectively. Only statistically significant PCC values were shown in the figure (t-test). (d) The number of interface and non-interface mutations. Interface residues were defined if any heavy atoms are within 5Å distance from any heavy atoms of ligand.

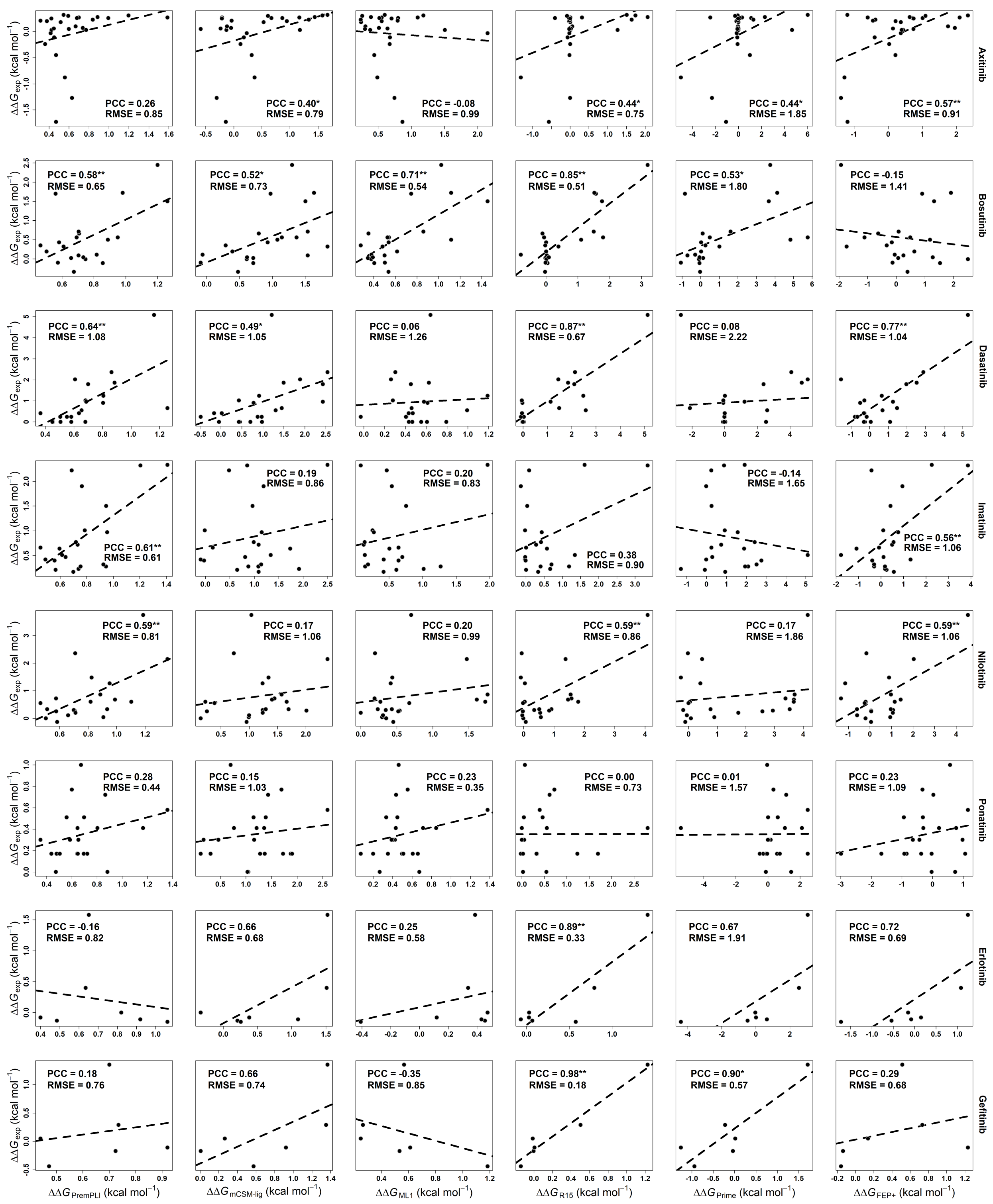

Supplementary Figure 6. Performance for six different methods tested on eight Abl-inhibitor complexes. * and ** indicate statistically significant difference from zero (t-test) in terms of PCC with p-value < 0.05 and p-value < 0.01, respectively.

a. PCC

	A14	A99	A99σ	C22	C36	C36m	A14+A99	A14+A99σ	A14+C22	A14+C36	A14+C36m	A99+A99σ	A99+C22	A99+C36	A99+C36m	A99σ+C22	A99σ+C36	A99σ+C36m	C22+C36	C22+C36m	C36+C36m
talaris2014	0.48	0.45	0.42	0.39	0.40	0.36	0.49	0.48	0.46	0.45	0.44	0.46	0.45	0.44	0.42	0.41	0.40	0.38	0.40	0.38	0.38
REF2015	0.45	0.42	0.40	0.36	0.37	0.33	0.47	0.46	0.44	0.43	0.42	0.44	0.43	0.42	0.40	0.39	0.38	0.37	0.38	0.36	0.36
beta_nov16	0.45	0.43	0.40	0.37	0.37	0.33	0.48	0.47	0.45	0.44	0.42	0.45	0.43	0.42	0.40	0.40	0.38	0.36	0.38	0.36	0.36

b. RMSE

	A14	A99	A99σ	C22	C36	C36m	A14+A99	A14+A99σ	A14+C22	A14+C36	A14+C36m	A99+A99σ	A99+C22	A99+C36	A99+C36m	A99σ+C22	A99σ+C36	A99σ+C36m	C22+C36	C22+C36m	C36+C36m
talaris2014	1.23	1.25	1.28	1.33	1.33	1.37	1.22	1.23	1.28	1.29	1.31	1.25	1.29	1.30	1.31	1.32	1.34	1.35	1.33	1.35	1.36
REF2015	1.26	1.28	1.29	1.35	1.35	1.39	1.24	1.24	1.29	1.30	1.32	1.26	1.31	1.31	1.33	1.33	1.34	1.36	1.35	1.37	1.37
beta_nov16	1.25	1.27	1.29	1.35	1.35	1.39	1.23	1.24	1.29	1.30	1.32	1.25	1.30	1.31	1.33	1.33	1.35	1.36	1.35	1.37	1.38

Supplementary Figure 7. Performance of combined Rosetta using three different scoring functions and MD approaches under six different force fields tested on S99 dataset. The PCC (a) and RMSE (b) were calculated using the predicted values taken from Aldeghi et al.[3] and the average values from Rosetta and MD approaches were used.

Supplementary Table 1. Experimental datasets used for training and testing different methods. S763 is the training set of mCSM-lig and S675 is the overlapped data between S796 and S763.

Dataset	# of mutations	# of complexes	# of ligands	# of proteins
**S796**	796	360	168	117
**S859**	859	360	168	117
**S144**	144	8	8	1
**S99**	99	42	22	14
**S763**	763	254	177	125
**S675**	675	240	166	116

Supplementary Table 2. The performance for PremPLI trained and tested on S859 and S796 dataset and performing four types of cross-validation, respectively.

Method	S859			S796		
	**PCC**	**RMSE**	**Slope**	**PCC**	**RMSE**	**Slope**
**PremPLI**	0.74	1.06	1.12	0.70	1.08	1.14
**PremPLI (CV1)**	0.73	1.07	1.14	0.69	1.10	1.17
**PremPLI (CV2)**	0.74	1.06	1.14	0.70	1.08	1.16
**PremPLI (CV3)**	0.51	1.34	0.96	0.55	1.26	1.01
**PremPLI (CV4)**	0.50	1.35	0.92	0.53	1.27	0.98

Pearson correlation coefficient between experimental and predicted $\Delta\Delta G$ values. RMSE (kcal mol^{-1}): root-mean-square error. Slope: the slope of the regression line between experimental and predicted $\Delta\Delta G$ values. All correlation coefficients are statistically significantly different from zero (p-value < 0.01, t-test).

Supplementary Table 3. The performance using Random Forest (RF), Support Vector Machine (SVM), eXtreme Gradient Boosting (XGBoost) and Extremely Randomized Trees (ExtraTrees) algorithms to build PremPLI model, respectively. Leave-one-complex-out results on S796 and the uncertainties in the measures of PCC RMSE and Slope are shown.

Algorithm	PCC	RMSE	Slope	PCC	RMSE	Slope
**RF**	0.55	1.26	1.01	$0.55_{0.49}^{0.60}$	$1.26_{1.16}^{1.35}$	$1.02_{0.89}^{1.14}$
**SVM**	0.43*	1.36	0.89	$0.43_{0.36}^{0.49}$	$1.36_{1.27}^{1.45}$	$0.90_{0.75}^{1.04}$
**XGBoost**	0.56	1.25	0.85	$0.57_{0.50}^{0.62}$	$1.25_{1.16}^{1.34}$	$0.85_{0.75}^{0.96}$
**ExtraTrees**	0.54	1.27	1.04	$0.54_{0.48}^{0.60}$	$1.27_{1.17}^{1.36}$	$1.04_{0.91}^{1.17}$

*p-value < 0.01 compared to Random Forest (Hittner2003 test).
In the bootstrap procedure, the PCC, RMSE and Slope of the three algorithms were significantly different from that of Random Forest (p-value < 0.01, t-test).

Supplementary Table 4. Performance on the test sets of S144, S129 and S99 using Random Forest (RF), Support Vector Machine (SVM), eXtreme Gradient Boosting (XGBoost) and Extremely Randomized Trees (ExtraTrees) algorithms to build PremPLI model, respectively. 95% bootstrapped confidence intervals are also shown for the PCC and RMSE. For S144 and S129, all algorithms were trained on S796; For S99, all algorithms were retrained after removing all mutations in the overlapped complexes with S99 from the training dataset.

**Dataset**	**Method**	**PCC**	**RMSE**	**PCC**	**RMSE**
**S144**	RF	0.46	0.77	$0.47_{0.31}^{0.60}$	$0.77_{0.63}^{0.95}$
	SVM	0.25*	0.84	$0.25_{0.04}^{0.43}$	$0.84_{0.69}^{1.01}$
	XGBoost	0.39	0.83	$0.39_{0.28}^{0.51}$	$0.83_{0.70}^{1.00}$
	ExtraTrees	0.33*	0.80	$0.34_{0.21}^{0.47}$	$0.80_{0.64}^{1.00}$
**S129**	RF	0.48	0.78	$0.48_{0.33}^{0.61}$	$0.77_{0.62}^{0.96}$
	SVM	0.20[a]*	0.89	$0.20_{-0.04}^{0.38}$	$0.89_{0.74}^{1.05}$
	XGBoost	0.36*	0.84	$0.36_{0.24}^{0.49}$	$0.84_{0.69}^{1.02}$
	ExtraTrees	0.32*	0.81	$0.33_{0.19}^{0.46}$	$0.81_{0.63}^{1.02}$
**S99**	RF	0.69	1.09	$0.68_{0.55}^{0.78}$	$1.09_{0.91}^{1.28}$
	SVM	0.47*	1.23	$0.46_{0.29}^{0.60}$	$1.23_{1.02}^{1.45}$
	XGBoost	0.67	1.06	$0.66_{0.48}^{0.78}$	$1.06_{0.87}^{1.27}$
	ExtraTrees	0.60*	1.17	$0.59_{0.43}^{0.71}$	$1.16_{0.97}^{1.37}$

All correlation coefficients are statistically significantly different from zero (p-value < 0.01, t-test) except [a]p-value = 0.02. * indicate statistically significant difference between PremPLI and other methods in terms of PCC (Hitter2003 test) with p-value < 0.01.
In the bootstrap procedure, the PCC and RMSE of the three algorithms were significantly different from that of Random Forest for all test sets (p-value < 0.01, t-test).

Supplementary Table 5. Comparison of methods' performances on the datasets of S144, S129 and S99. 95% bootstrapped confidence intervals are shown for all performance measures.

Dataset	Method	PCC	RMSE	AUC-ROC	AUC-PR	MCC
**S144**	PremPLI	$0.47_{0.31}^{0.60}$	$0.77_{0.63}^{0.95}$	$0.78_{0.67}^{0.88}$	$0.38_{0.18}^{0.59}$	$0.45_{0.25}^{0.65}$
	mCSM-lig	$0.42_{0.30}^{0.54}$	$0.91_{0.78}^{1.06}$	$0.75_{0.65}^{0.85}$	$0.32_{0.16}^{0.51}$	$0.39_{0.25}^{0.56}$
	ML1	$0.13_{-0.04}^{0.30}$	$0.87_{0.69}^{1.08}$	$0.61_{0.45}^{0.75}$	$0.21_{0.11}^{0.35}$	$0.27_{0.10}^{0.44}$
	R15	$0.66_{0.45}^{0.81}$	$0.72_{0.61}^{0.84}$	$0.76_{0.59}^{0.90}$	$0.49_{0.27}^{0.68}$	$0.55_{0.38}^{0.70}$
	Prime	$0.30_{0.04}^{0.52}$	$1.81_{1.48}^{2.15}$	$0.67_{0.52}^{0.81}$	$0.28_{0.14}^{0.48}$	$0.39_{0.19}^{0.59}$
	FEP+	$0.48_{0.22}^{0.69}$	$1.06_{0.88}^{1.24}$	$0.76_{0.59}^{0.90}$	$0.52_{0.29}^{0.72}$	$0.56_{0.37}^{0.74}$
**S129**	PremPLI	$0.48_{0.33}^{0.61}$	$0.77_{0.62}^{0.96}$	$0.81_{0.71}^{0.89}$	$0.36_{0.18}^{0.55}$	$0.44_{0.28}^{0.61}$
	mCSM-lig	$0.20_{-0.10}^{0.50}$	$1.05_{0.79}^{1.35}$	$0.54_{0.36}^{0.71}$	$0.28_{0.11}^{0.51}$	$0.41_{0.14}^{0.63}$
**S99**	PremPLI	$0.68_{0.55}^{0.78}$	$1.09_{0.91}^{1.28}$	$0.84_{0.70}^{0.94}$	$0.69_{0.50}^{0.84}$	$0.70_{0.52}^{0.85}$
	A14	$0.44_{0.25}^{0.58}$	$1.35_{1.16}^{1.55}$	$0.78_{0.62}^{0.91}$	$0.54_{0.30}^{0.73}$	$0.58_{0.38}^{0.78}$
	R14	$0.33_{0.18}^{0.46}$	$1.34_{1.15}^{1.55}$	$0.68_{0.54}^{0.82}$	$0.39_{0.19}^{0.58}$	$0.41_{0.22}^{0.60}$
	RMD	$0.48_{0.32}^{0.59}$	$1.22_{1.05}^{1.42}$	$0.77_{0.62}^{0.91}$	$0.57_{0.35}^{0.76}$	$0.60_{0.40}^{0.78}$

The PCC, RMSE, AUC-ROC, AUC-PR and MCC of all methods to be compared were significantly different from that of PremPLI (p-value < 0.01, t-test).

Supplementary Table 6. The performance using Random Forest (RF), eXtreme Gradient Boosting (XGBoost) and Extremely Randomized Trees (ExtraTrees) algorithms to build PremPLI model, respectively. Leave-one-complex-out results on S144 and the uncertainties in the measures of PCC RMSE and Slope are shown.

Algorithm	PCC	RMSE	Slope	PCC	RMSE	Slope
**RF**	0.70	0.60	1.23	$0.70_{0.54}^{0.81}$	$0.60_{0.50}^{0.72}$	$1.23_{0.87}^{1.58}$
**XGBoost**	0.73	0.57	1.00	$0.72_{0.60}^{0.82}$	$0.57_{0.47}^{0.68}$	$1.00_{0.74}^{1.28}$
**ExtraTrees**	0.72	0.60	1.31	$0.71_{0.57}^{0.81}$	$0.60_{0.49}^{0.72}$	$1.31_{0.95}^{1.69}$

In the bootstrap procedure, the PCC, RMSE, and Slope of the two algorithms were significantly different from that of Random Forest (p-value < 0.01, t-test).

Supplementary Table 7. Computation time required for each feature for a single mutation per protein with ~ 400 residues and each additional mutation introduced in the same complex on a single CPU core. For each additional mutation, most time is taken up by FoldX to construct the mutant structure.

Feature	A single mutation	Each additional mutation
$\boldsymbol{\Delta CS}$	15s	0.01s
$\boldsymbol{MW}$t	0.5s	0s
$\boldsymbol{Prox^{wt}}$	5s	0s
$\boldsymbol{PSSM}$	9min30s	0s
$\boldsymbol{P_{RKDE}}$	0.5s	0s
$\boldsymbol{P_Q}$	0.5s	0s
$\boldsymbol{N_{NO}}$	0.5s	0s
$\boldsymbol{H}$	0.01s	0s
$\boldsymbol{M_{AA1}}$	0.01s	0.01s
$\boldsymbol{Prox^{mut}}$	20s	20s
$\boldsymbol{M_{AA2}}$	0.01s	0.01s
**Total**	10min12s	20.03s

Supplementary Table 8. Computational cost for different approaches. The data for ML1, FEP+, and R15 were taken from Aldeghi et al.[4].

Abbreviation	Method	Force filed or scoring function	Approximate cost per $\Delta\Delta G$ calculation	
			**Hardware**	**Compute hours**
PremPLI	Machine Learning	n/a	1 CPU core	0.17
ML1	Machine Learning	n/a	1 CPU core	0.02
FEP+	Molecular Dynamics	OPLS3	1 GPU	72
R15	Rosetta	REF15	1 CPU core	32

Supplementary Table 9. Features considered in model selection.

**Features**
**Sequence-based features**
Amino acid substitution indexes and statistical protein contact potentials from AAindex database.
The length of protein and mutated chain
Physicochemical properties of mutations and residues around the mutation site
Configurational entropy of mutated residue
Hydrophobicity of mutated residue
Conservation score of the mutated site
Relative molecular mass of the mutated site
Volume of the mutated site
Proportion of different types of residues in the mutated chain
**Structure-based features**
Solvent accessible surface area
Energy terms from FoldX
The proportion of different residues classified by buried, moderately buried and exposed in the whole protein structure
Secondary structure elements
Non-covalent interactions between protein and ligand
Protein-ligand binding affinity
The number of residues in contact with the ligands
Ligand descriptors including molecular weight, number of H-bond donors and acceptors, oxygen and nitrogen atoms, rotatable bonds and rings, and logP.

Supplementary Table 10. The importance of each feature in random forest scoring function of PremPLI. IncNodePurity is used for describing the importance which is the total decrease in node impurities from splitting on the variable, averaged over all trees.

Feature	Importance
$\boldsymbol{\Delta CS}$	371
$\boldsymbol{MW}$t	164
$\boldsymbol{Prox^{wt}}$	148
$\boldsymbol{PSSM}$	142
$\boldsymbol{P_{RKDE}}$	137
$\boldsymbol{P_{Q}}$	134
$\boldsymbol{N_{NO}}$	132
$\boldsymbol{H}$	125
$\boldsymbol{M_{AA1}}$	113
$\boldsymbol{Prox^{mut}}$	112
$\boldsymbol{M_{AA2}}$	96

Supplementary References

1. Hauser K*, et al.* Predicting resistance of clinical Abl mutations to targeted kinase inhibitors using alchemical free-energy calculations. *Communications Biology* **1**, 70 (2018).

2. Jubb HC, Higueruelo AP, Ochoa-Montano B, Pitt WR, Ascher DB, Blundell TL. Arpeggio: A Web Server for Calculating and Visualising Interatomic Interactions in Protein Structures. *Journal of Molecular Biology* **429**, 365-371 (2017).

3. Aldeghi M, Gapsys V, de Groot BL. Accurate Estimation of Ligand Binding Affinity Changes upon Protein Mutation. *ACS Central Science* **4**, 1708-1718 (2018).

4. Aldeghi M, Gapsys V, de Groot BL. Predicting Kinase Inhibitor Resistance: Physics-Based and Data-Driven Approaches. *ACS Central Science* **5**, 1468-1474 (2019).
